# Effectiveness of laparoscopic removal of isolated superficial peritoneal endometriosis for the management of chronic pelvic pain in women (ESPriT2): protocol for a multi-centre randomised controlled trial

**DOI:** 10.1186/s13063-023-07386-x

**Published:** 2023-06-22

**Authors:** Scott C. Mackenzie, Jacqueline Stephen, Linda Williams, Jane Daniels, John Norrie, Christian M. Becker, Dominic Byrne, Ying Cheong, T. Justin Clark, Kevin G. Cooper, Emma Cox, Ann M. Doust, Priscilla Fernandez, Jeremy Hawe, Tom Holland, Lone Hummelshoj, Louise J. Jackson, Kathleen King, Abha Maheshwari, Dan C. Martin, Lauren Sutherland, Jim Thornton, Katy Vincent, Sanjay Vyas, Andrew W. Horne, Lucy H. R. Whitaker

**Affiliations:** 1 MRC Centre for Reproductive Health, University of Edinburgh, Edinburgh, EH16 4TJ UK; 2 Edinburgh Clinical Trials Unit, Usher Institute, University of Edinburgh, NINE, Edinburgh BioQuarter, Edinburgh, EH16 4UX UK; 3 Nottingham Clinical Trials Unit, University of Nottingham, Nottingham, NG7 2RD UK; 4 Endometriosis CaRe, Nuffield Department of Women’s and Reproductive Health, University of Oxford, Oxford, OX3 9DU UK; 5 Royal Cornwall Hospitals NHS Trust, Truro, UK; 6 Faculty of Medicine, Human Development and Health, University of Southampton, Southampton, UK; 7 Birmingham Women’s and Children Hospital, Birmingham, B15 2TG UK; 8 NHS Grampian, Aberdeen Royal Infirmary, Foresterhill, Aberdeen, AB25 2ZN UK; 9 Endometriosis UK, London, UK; 10 Corniche Hospital, Abu Dhabi, United Arab Emirates; 11 Guys and St Thomas NHS Trust, London, UK; 12 Endometriosis.org, London, UK; 13 University of Birmingham, Birmingham, UK; 14 Letterkenny, Ireland; 15 Aberdeen Fertility Centre, NHS Grampian, Aberdeen, UK; 16 Department of Obstetrics and Gynecology, University of Tennessee Health Science Center, Memphis, TN USA; 17 Virginia Commonwealth University, Institutional Review Board, Richmond, VA USA; 18 EndoFound (Endometriosis Foundation of America), New York, USA; 19 University of Nottingham, Nottingham, UK; 20 Southmead Hospital, North Bristol NHS Trust, Bristol, BS10 5NB UK

**Keywords:** Endometriosis, Pelvic pain, Laparoscopy, Surgical ablation, Surgical excision, Randomised controlled trial

## Abstract

**Background:**

Endometriosis affects 190 million women and those assigned female at birth worldwide. For some, it is associated with debilitating chronic pelvic pain. Diagnosis of endometriosis is often achieved through diagnostic laparoscopy. However, when isolated superficial peritoneal endometriosis (SPE), the most common endometriosis subtype, is identified during laparoscopy, limited evidence exists to support the common decision to surgically remove it via excision or ablation. Improved understanding of the impact of surgical removal of isolated SPE for the management of chronic pelvic pain in women is required. Here, we describe our protocol for a multi-centre trial to determine the effectiveness of surgical removal of isolated SPE for the management of endometriosis-associated pain.

**Methods:**

We plan to undertake a multi-centre participant-blind parallel-group randomised controlled clinical and cost-effectiveness trial with internal pilot. We plan to randomise 400 participants from up to 70 National Health Service Hospitals in the UK. Participants with chronic pelvic pain awaiting diagnostic laparoscopy for suspected endometriosis will be consented by the clinical research team. If isolated SPE is identified at laparoscopy, and deep or ovarian endometriosis is not seen, participants will be randomised intraoperatively (1:1) to surgical removal (by excision or ablation or both, according to surgeons’ preference) versus diagnostic laparoscopy alone. Randomisation with block-stratification will be used. Participants will be given a diagnosis but will not be informed of the procedure they received until 12 months post-randomisation, unless required. Post-operative medical treatment will be according to participants’ preference. Participants will be asked to complete validated pain and quality of life questionnaires at 3, 6 and 12 months after randomisation. Our primary outcome is the pain domain of the Endometriosis Health Profile-30 (EHP-30), via a between randomised group comparison of adjusted means at 12 months. Assuming a standard deviation of 22 points around the pain score, 90% power, 5% significance and 20% missing data, 400 participants are required to be randomised to detect an 8-point pain score difference.

**Discussion:**

This trial aims to provide high quality evidence of the clinical and cost-effectiveness of surgical removal of isolated SPE.

**Trial registration:**

ISRCTN registry ISRCTN27244948. Registered 6 April 2021.

## Administrative information

Note: the numbers in curly brackets in this protocol refer to SPIRIT checklist item numbers. The order of the items has been modified to group similar items (see http://www.equator-network.org/reporting-guidelines/spirit-2013-statement-defining-standard-protocol-items-for-clinical-trials/).

**Table:** 

Title {1}	Effectiveness of laparoscopic removal of isolated superficial peritoneal endometriosis for the management of chronic pelvic pain in women (ESPriT2): protocol for a multi-centre randomised controlled trial
Trial registration {2a and 2b}.	ISRCTN registry ISRCTN27244948. Registered 6th April 2021.
Protocol version {3}	Version 4.0
Funding {4}	This study is funded by the NIHR Health Technology Assessment programme (NIHR129801). The views expressed are those of the author(s) and not necessarily those of the NIHR or the Department of Health and Social Care.
Author details {5a}	**SCM, AMD, PF, LS, AWH, LHRW:** MRC Centre for Reproductive Health, University of Edinburgh, Edinburgh, EH16 4TJ, UK **JS, LW, JN:** Usher Institute, Edinburgh Clinical Trials Unit, University of Edinburgh, NINE, Edinburgh BioQuarter, Edinburgh, EH16 4UX, UK **JD:** Nottingham Clinical Trials Unit, University of Nottingham, Nottingham, NG7 2RD, UK **KV, CMB:** Endometriosis CaRe, Nuffield Department of Women’s and Reproductive Health, University of Oxford, Oxford, OX3 9DU, UK **DB:** Royal Cornwall Hospitals NHS Trust, Truro, UK **YC:** Faculty of Medicine, Human Development and Health, University of Southampton, Southampton, UK **TJC:** Birmingham Women's and Children Hospital, Birmingham, B15 2TG, UK **KGC:** NHS Grampian, Aberdeen Royal Infirmary, Foresterhill, Aberdeen, AB25 2ZN, UK **EC:** Endometriosis UK **JH:** Corniche Hospital, Abu Dhabi, United Arab Emirates. **TH:** Guys and St Thomas NHS Trust, London, UK **LH:** Endometriosis.org **LJJ:** University of Birmingham, Birmingham, UK **KK:** Independent endometriosis advocate, Ireland. **AM:** Aberdeen Fertility Centre, NHS Grampian, Aberdeen, UK **DCM:** University of Tennessee Health Science Center, Department of Obstetrics and Gynecology, Memphis, Tennessee, USA; Virginia Commonwealth University, Institutional Review Board, Richmond, Virginia, USA; Scientific and Medical Director of EndoFound (Endometriosis Foundation of America) **JT:** University of Nottingham, Nottingham, UK **SV:** Southmead Hospital, North Bristol NHS Trust, Bristol BS10 5NB
Name and contact information for the trial sponsor {5b}	University of Edinburgh & NHS Lothian ACCORD Research Governance & QA Office, The Queen’s Medical Research Institute, 47 Little France Crescent Edinburgh, EH16 4TJ, Email: resgov@accord.scot
Role of sponsor {5c}	The sponsor is the individual, organisation or partnership that takes on overall responsibility for proportionate, effective arrangements being in place to set up, run and report a research project.

## Introduction

### Background and rationale {6a}

Endometriosis is a chronic oestrogen-dependent, neuro-inflammatory condition that affects approximately 10% of women and those assigned female at birth of reproductive age worldwide [1]. It is characterized by the growth of endometrial-like tissue (‘lesions’) outside of the uterus, most commonly on the peritoneum and ovaries. Symptoms can include chronic pelvic pain, painful periods, painful sex, infertility and fatigue [2]. Endometriosis-associated pain can be disabling and worsen quality of life; the societal economic cost is estimated at £8500 per woman per year globally [3].

Three subtypes of endometriosis have been described: superficial peritoneal endometriosis (SPE), ovarian endometriosis and deep endometriosis [2]. Around 80% of those with endometriosis have SPE. A lack of non-invasive endometriosis tests mean definitive diagnosis is usually achieved by visualisation of the lesions at diagnostic laparoscopy [2]. During laparoscopy, if SPE is found, gynaecologists usually remove it surgically (by excision or ablation) [4, 5]. Investigation and surgical removal require gynaecological surgical expertise and form a significant proportion of the gynaecological workload. However, around 50% of patients who have undergone surgical treatment for endometriosis re-present with persistent, or recurrent, pain within 5 years and surgical reintervention rates are high [6, 7].

Limited evidence exists to support current endometriosis guidelines, showing whether or not surgical removal of isolated SPE improves (or worsens) symptoms and quality of life [4, 5]. A 2014 Cochrane systematic review and meta-analysis of laparoscopic surgery for endometriosis concluded that laparoscopic treatment leads to improved condition-associated pain (cited as ‘better’ or ‘improved’) when compared to diagnostic laparoscopy alone at 6 months (OR 6.58, 95% CI 3.31–13.10) [8]. However, this conclusion is drawn from three randomised controlled trials (RCT), comprising 171 participants with multiple endometriosis subtypes. Furthermore, only one small RCT included in the analysis (69 participants) has follow-up data to 12 months showing benefit of surgery (OR 10.00, 95% CI 3.21–31.17). This led the authors of the systematic review to define the strength of the evidence, based on GRADE criteria, as of moderate and low quality at six and 12 months respectively.

This Cochrane review was updated in 2020, but despite reviewing 1175 articles, the authors reduced the number of included trials examining the effect of laparoscopic treatment of endometriosis on pain to one study of 16 participants with mixed disease type [9]. This was a consequence of altered methodology including application the core outcome set for trial reporting in endometriosis studies [10]. Whilst the authors noted that surgery improved pain at 12 months, the study was considered ‘very low-quality’ evidence. This led the authors to conclude that they were ‘uncertain of the effect of laparoscopic excision on overall pain scores compared to diagnostic laparoscopy only and that further trials are needed’. The paucity of evidence and need for additional studies was further echoed by an additional systematic review and meta-analysis of the effectiveness of laparoscopic surgery for endometriosis, also published in 2020 [11]. Furthermore, the uncertainty surrounding the effectiveness of laparoscopic management of SPE is compounded by the limited evidence to allow an informed selection of specific surgical modalities to remove SPE (ablation versus excision) [12].

Consequently, both the 2017 National Institute of Clinical Excellence (NICE) Endometriosis Guideline and the recent Endometriosis Guideline by the European Society of Human Reproduction and Embryology (ESHRE) recommend further research is needed to determine the effectiveness of laparoscopic removal of isolated SPE to manage endometriosis-associated pain [4, 5]. This call for research was further supported by the outcome of the 2017 James Lind Alliance Endometriosis Priority Setting Partnership that established key research questions most important to those with endometriosis and healthcare practitioners [13].

We, therefore, plan to undertake a multi-centre trial across the UK (called ESPriT2) where women who have only SPE found at diagnostic laparoscopy are randomly allocated to have surgical removal of SPE or diagnostic laparoscopy alone. Surgical removal of SPE in the context of this trial means excision, ablation, or a combination of both. We aim to determine whether surgical removal of endometriotic lesions improves overall endometriosis-associated symptoms and quality of life or whether surgery is of no benefit, exacerbates symptoms or even causes harm.

In this protocol, from now on we use the terms ‘woman’ and ‘women.’ However, it is important to note that endometriosis can affect all people assigned female at birth, all of whom are eligible to participate in this trial.

### Objectives {7}

The primary objective of this trial is to compare laparoscopic removal of isolated SPE versus diagnostic laparoscopy alone in terms of participants’ pain at 12 months post-randomisation.

The secondary objectives of this trial are to compare laparoscopic removal of isolated SPE versus diagnostic laparoscopy alone in terms of:

- Physical and emotional functioning
- Requirement for future intervention
- Occupational outcomes
- Post-operative pain scores
- Surgical complications
- Pregnancy events
- Cost-effectiveness
- Adverse events

### Trial design {8}

**Fig. 1:**
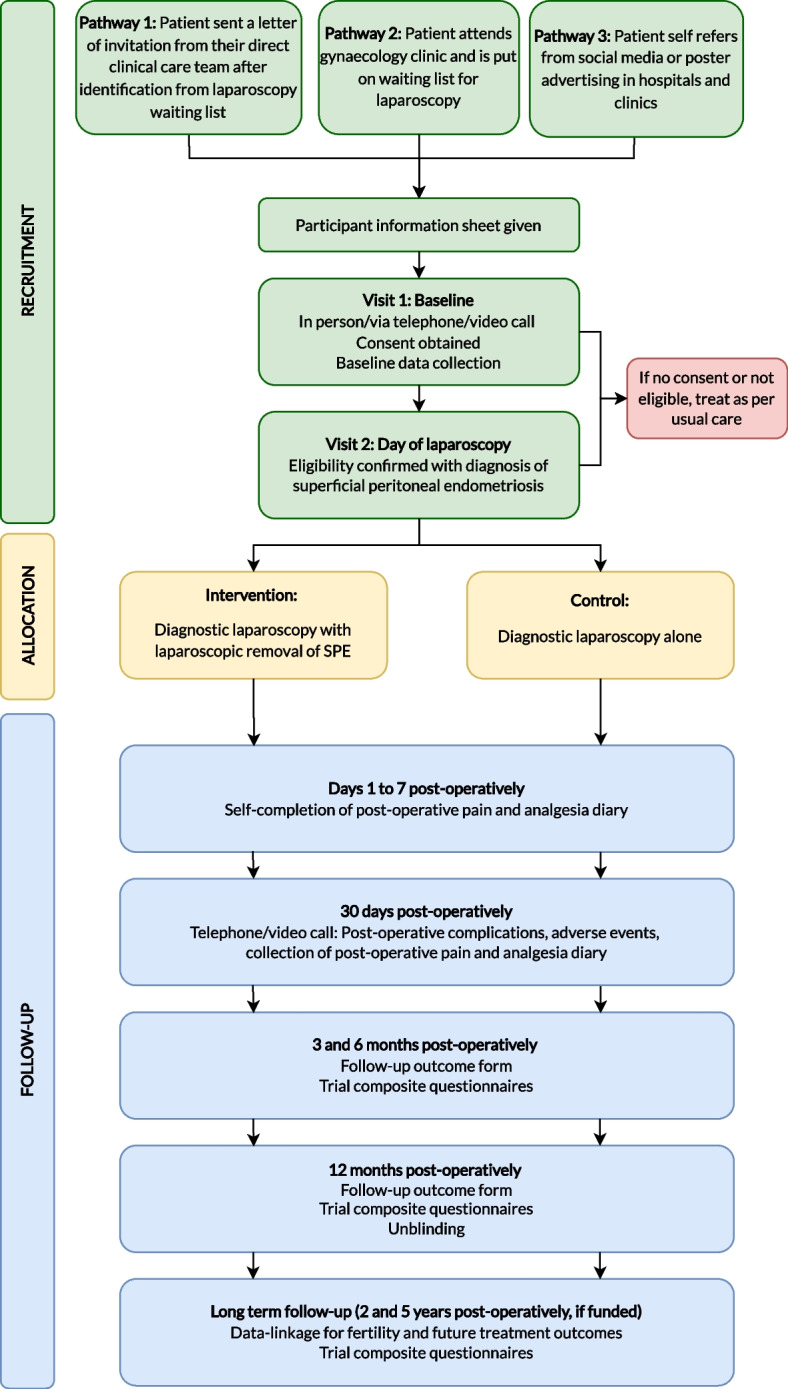
Schematic diagram of ESPriT2 trial design

ESPriT2 is a multi-centre participant-blind parallel-group, superiority randomised (1:1) controlled clinical and cost-effectiveness trial with internal pilot. Figure 1 provides a schematic diagram of the ESPriT2 trial design. The trial design and development of patient facing materials was informed by an ESPriT2 patient and public involvement panel consisting of individuals with lived experience of endometriosis and representatives from patient support organisations.

## Methods: participants, interventions and outcomes

### Study setting {9}

This trial will take place in National Health Service (NHS) hospitals in the UK including both British Society for Gynaecological Endoscopy (BSGE) centres and general gynaecology units. We aim to recruit in up to 70 hospital sites.

### Eligibility criteria {10}

Inclusion and exclusion criteria for trial participants are detailed in Table 1.

**Table 1:** Inclusion and exclusion criteria for trial participants

Inclusion criteria	Exclusion criteria
• Women* aged over 16 • Undergoing laparoscopy for the investigation of chronic pelvic pain • In order to be randomised, isolated superficial peritoneal endometriosis (SPE) must be identified at laparoscopy (macroscopically) • Able to give informed consent	• Previous surgical diagnosis of endometriosis • Pregnant • Women* who have undergone hysterectomy and/or bilateral oophorectomy • Women* who are undergoing the following concurrent procedures at the time of laparoscopy o Salpingectomy o Ovarian cystectomy o Oophorectomy o Division of dense adhesions o Endometrial ablation • Ovarian cyst on imaging that is the indication for surgery • Deep endometriosis on imaging or at time of laparoscopy • Endometrioma observed at the time of laparoscopy • Peritoneal ‘pockets’ only noted at laparoscopy

^*^Women includes those assigned female at birth

### Who will take informed consent? {26a}

Potentially eligible participants who are referred or self-refer will be provided with a patient information sheet by a member of the clinical research team at their trial site. Initial contact may be face-to-face during a routine clinic appointment or may be via a telephone/video call via an approved NHS platform by a member of the research team.

Informed consent to participate in the trial will only to be taken by a member of the clinic research team once the patient has had sufficient time to read the patient information sheet, have their questions answered and consider whether they wish to participate in the trial. At least 24 h will be provided for potentially eligible participants to consider participation. All participants who are approached via a call will have the option to attend the hospital to discuss the trial and provide written informed consent in person or provide informed consent verbally over the call. Those who give verbal consent will have the consent form signed by the researcher and a copy of this signed form will be sent to the participant with contact details of the research team should they decide to withdraw consent. At the time of consent, the participant will be reminded that they will be given a diagnosis post-operatively of the findings at the time of laparoscopy but will not be told if surgical removal was carried out. All participants will be asked to re-confirm consent at the time of their laparoscopy and will be asked to wet ink sign the trial consent form before any further research activities are carried out although questionnaires may be completed before this signature is obtained.

### Additional consent provisions for collection and use of participant data and biological specimens {26b}

Participants consented to ESPriT2 will be offered the option to participate in related biomarker (venous blood sample prior to surgery) and imaging (pelvic ultrasound) studies (ESPriT +) for future analysis. In addition, consent will be obtained for longer-term follow-up at two and five years and data linkage for fertility outcomes and surgical reintervention.

## Interventions

### Explanation for the choice of comparators {6b}

Diagnostic laparoscopy is required to diagnose SPE, compared to other endometriosis subtypes (ovarian or deep) that can sometimes be diagnosed using ultrasound by a skilled operator. When SPE is identified, management options include surgical removal (including surgical excision or surgical ablation or both) or no operative treatment. The term surgical removal of SPE in the context of this trial means excision, ablation or a combination of both.

### Intervention description {11a}

At the time of diagnostic laparoscopy, participants will be randomised to diagnostic laparoscopy with surgical removal of SPE or diagnostic laparoscopy alone. For participants allocated to surgical removal of SPE, excision and/or ablation of SPE will be dependent on the operating surgeon’s preference/usual practice. To maintain participant blinding, all participants will have two accessory ports sited in addition to the optical (usually umbilical) port. All participants will be informed of a diagnosis post-operatively but will not be informed whether surgical removal of SPE occurred.

### Criteria for discontinuing or modifying allocated interventions {11b}

Allocation will occur during diagnostic laparoscopy once eligibility (see Table 1) has been confirmed. Perioperative complications or surgical complexity may lead to discontinuation of the allocated intervention, and this is at the discretion of the operating surgeon. Reasons for incomplete removal of disease in those randomised to removal are documented at the end of the procedure if applicable.

### Strategies to improve adherence to interventions {11c}

Not applicable. The trial intervention will occur whilst the patient is anaesthetised.

### Relevant concomitant care permitted or prohibited during the trial {11d}

All participants will be offered routine NHS gynaecological care during the trial, which includes the option of taking any of the current recommended treatment options for endometriosis, such as oral analgesia or hormones (including concurrent levonorgestrel-intrauterine system insertion at surgery). Participants will be permitted to take part in non-interventional studies such as questionnaire studies and bio banking (with the exception of peritoneal biopsies). Participants will be discouraged from co-enrolling in other drug, treatment of pain or surgical trials. If participants co-enrol in another drug, treatment of pain or surgical trial their subsequently collected ESPriT2 data will be excluded from our analysis. Similarly, those enrolled in active intervention phase of another gynaecological trial will be excluded but eligible for inclusion if in long term follow-up phase of other gynaecological trials.

### Provisions for post-trial care {30}

At the end of 12 months of post-randomisation follow-up, participants will be given the option to be told which group they were randomised to. If they were allocated to the diagnostic laparoscopy alone group, they will have the opportunity to discuss whether they wish to have surgical removal of SPE as a component of their routine NHS gynaecological follow-up.

### Outcomes {12}

The primary outcome in this trial is the ‘pain domain’ of the Endometriosis Health Profile-30 (EHP-30) questionnaire at 12 months post-randomisation.

EHP-30 is a commonly used, condition-specific, patient reported outcome questionnaire that assesses health-related quality of life in endometriosis across five key domains (pain, control and powerlessness, emotional well-being, social support, and self-image) [14]. Evaluation of EHP-30 has shown consistent acceptability, validity, reliability and sensitivity to change suitable for use within endometriosis research and clinical trials of endometriosis interventions [15, 16].

Secondary clinical outcomes will include measures of use of hormones and analgesics, occupational impact, post-operative pain and reproductive outcomes. Secondary outcomes are detailed as follows:

- Time off work and presenteeism defined by the Work Productivity and Activity Impairment Questionnaire (WPAIQ) at 12 months
- Need for hormonal medication for endometriosis-related symptoms at 3, 6 and 12 months
- Need for analgesics for endometriosis-related symptoms at 3, 6 and 12 months
- Pain domain of the EHP-30 at 3 and 6 months
- Total score of EHP-30 at 3, 6 and 12 months
- Fatigue symptoms defined by the Brief Pain Inventory (BFI) at 12 months
- Neuropathic pain features identified by PainDETECT™ at 12 months
- Urinary symptoms defined by Pelvic Pain and Urinary Frequency (PUF) Patient Symptom Scale at 12 months
- Irritable bowel symptoms defined by the ROME IV Criteria at 12 months
- Pain catastrophizing measured by Pain Catastrophizing Questionnaire (PCQ) at 12 months
- Fibromyalgia defined by Fibromyalgia Scale (FS) at 12 months
- Specific patient reported symptoms defined by Measure Yourself Medical Outcome Profile 2 (MYMOP2)
- Post-operative (up to 7 days after surgery) pain and analgesic requirements by patient reported diary
- Length of hospital stay
- Adverse events related to surgery at 30 days
- Surgical complications at 30 days
- Surgical operating time
- Need for further surgery for endometriosis related symptoms at 12 months
- Pregnancy events at 3, 6 and 12 months

Outcomes used for the economic evaluation will include:

- Utility values, derived from EQ-5D-5L health-related quality of life score at 3, 6, and 12 months
- General wellbeing defined by Capability Questionnaire (ICECAP-A) at 3, 6, and 12 months
- Costs and resource use at 3, 6 and 12 months (primary and secondary care use)
- Impacts on employment, caregiving, and other usual activities (e.g. education)

For those allocated to surgical removal, we will also report the operative technique for removal of endometriosis (excision, ablation, or combination) and adequacy of removal of endometriosis (subjective and independent). Subject to further funding we aim to compare future fertility and need for further surgery using data linkage and assess EHP-30, ROME IV, PUF, EQ-5D-5L, PainDETECT™, hormonal and analgesic use, fertility interventions and pregnancy events measured at 2 and 5 years post-randomisation.

### Participant timeline {13}

A schedule of events for participants is detailed in Table 2.

**Table 2:** Trial composite questionnaires can be completed in person, via telephone, via post or via online link (*). Follow-up outcome questions are completed via a telephone call (**). Consent confirmed if initial consent was via telephone (***). NB: Longer term follow-up not shown in this table as not funded at time of writing

Phase	Baseline	Randomisation (day of surgery)	Days 1–7 post-operatively (± 1 week)	30 days post-operatively (± 1 week)	3 months post-operatively (± 2 weeks)	6 months post-operatively (± 2 weeks)	12 months post-operatively (± 2 weeks)
Consent to trial	**x**	x***					
Eligibility	**x**	**x**					
Medical history	**x**						
Post-operative pain diary			**x**				
Post-operative phone call (complications)				**x**			
Trial composite questionnaires*	**x**				**x**	**x**	**x**
Follow-up outcome form**					**x**	**x**	**x**
Adverse events				**x**			

### Sample size {14}

An 8-point difference of the EHP-30 pain domain is equivalent to a standardized difference of 0.36 (assuming a standard deviation of 22) and reduction in pain of this magnitude is considered clinically significant. Data from a UK endometriosis trial (PRE-EMPT ISRCTN97865475) shows a standard deviation (SD) of 19 (95% CI 16–22) in baseline EHP-30 scores [17]. Assuming an 8-point difference and a SD of 22, 160 participants are required in each treatment arm to detect that difference with 90% power and a two-sided 5% significance level. Assuming a maximum of 20% loss of primary outcome data, 400 randomised participants will be required. We acknowledge that the variability in our outcome may be different to that observed in the PRE-EMPT trial due to differences in the intervention; therefore, the ESPriT2 trial steering committee (TSC) will review blinded pooled EHP-30 data and advise whether the sample size should be reconsidered, if necessary.

### Recruitment {15}

We will randomise 400 participants (200 per trial group). To achieve this, we estimate we will need to consent 1000–1200 participants (based on our experience of recruiting to similar surgical endometriosis trials). Identification of potentially eligible ESPriT2 participants will occur via three pathways. Potentially eligible participants can be identified via gynaecology out-patient departments following a clinical decision to perform a diagnostic laparoscopy for the investigation of chronic pelvic pain. Alternatively, potentially eligible participants can be identified from diagnostic laparoscopy waiting lists and invited to participate, or individuals can self-refer after seeing trial promotional material.

## Assignment of interventions: allocation

### Sequence generation {16a}

Eligibility will be confirmed at the time of laparoscopy following a finding of SPE only. Participants will be randomised in 1:1 ratio to either diagnostic laparoscopy alone or to concurrent surgical removal (ablation/excision/combination of both) using a remote randomisation system provided independently by Edinburgh Clinical Trials Unit (ECTU). Randomisation will use a computer-based randomisation system stratified using permuted blocks by the following important prognostic variables:

- Presence of dysmenorrhoea (yes/no)
- Pre-operative hormone treatment (yes/no)
- Presence of dyspareunia (yes/no)

### Concealment mechanism {16b}

The participant’s intraoperative data will be entered into a 24-h computerised central randomisation service by means of a secure web interface or by a telephone call to the trial management team.

### Implementation {16c}

The ECTU randomisation service determines the treatment allocation and will be given to the operating surgeon. If the database is not available, emergency randomisations will be performed by simple randomisation using a computer-generated random number list provided independently by ECTU. The allocation sequence will be enclosed in sequentially numbered, opaque, sealed envelopes. Envelopes are opened by the research team only after the enrolled participant has eligibility confirmed, and the treatment allocation will be given to the operating surgeon.

## Assignment of interventions: blinding

### Who will be blinded {17a}

This is a participant blind trial. Participants will be informed of a diagnosis post-operatively but will not be informed if surgical removal was carried out. All attempts will be made to minimise the inadvertent unblinding of all trial participants. This will include providing templates for operative findings and standardised discharge letters. The operation note will include only the operative findings but not treatment allocation or details of surgical removal (if applicable). These will be recorded within the surgical case report form (SCRF) and will be added to the individuals NHS records at the end of participation. Participants’ general practitioners will not be informed of whether surgical removal of SPE occurred during the trial period but will be informed when a diagnosis of SPE is made. Where feasible, the onward clinical management within secondary care will not be those who performed surgery. The importance of maintaining blinding will be emphasised to all members of the surgical care team, e.g. anaesthetic staff, theatre, and recovery staff, etc.

### Procedure for unblinding if needed {17b}

At the end of 12 months of post-randomisation follow-up, participants will be given the option to be unblinded. An unblinding facility will be available on the database. If it becomes necessary to unblind a participant prior (e.g. at the participant’s request or for emergency purposes), they will need to contact the local principal investigator (PI) and local research team who will follow the unblinding procedure on the trial database. This will be documented on the database with the reason why the unblinding has taken place as well as the date of the unblinding. Participants unblinded prior to 12 months will still be requested to complete trial outcome measures.

## Data collection and management

### Plans for assessment and collection of outcomes {18a}

#### Trial composite questionnaire

The trial composite questionnaire contains a range of validated patient-reported outcome measures. Responses to this questionnaire will be collected pre-operatively and then at 3, 6 and 12 months post-operatively (see Table 2). It can be completed in person, via telephone, via post or via online link. The baseline and follow-up trial composite questionnaire will include:

- Endometriosis Health Profile-30 (EHP-30) [14]
  o 30 question and five scale endometriosis questionnaire (explained above)
- Rome IV Criteria [18]
  o Three questions to identify irritable bowel syndrome
- Pelvic Pain and Urinary Frequency (PUF) Patient Symptom Scale [19]
  o Eight-point scale about urinary patterns and pain
- PainDETECT.™ [20]
  o 14-item questionnaire to identify neuropathic pain
- Brief Fatigue Inventory (BFI) [21]
  o Six-item questionnaire correlated with standard quality of life measures assessing severity of fatigue and impact of fatigue on functioning over the previous 24 h
- Pain Catastrophizing Questionnaire (PCQ) [22]
  o 13-item scale, with each item rated on a 5-point scale. The PCQ is broken into three subscales being magnification, rumination, and helplessness. The scale was developed as a self-report measurement tool that provided a valid index of catastrophising
- Fibromyalgia Scale [23]
  o Seven questions related to fibromyalgia symptoms
- Measure Yourself Medical Outcome Profile 2 (MYMOP2) [24]
  o Patient-generated outcome questionnaire
- Work Productivity and Activity Impairment Questionnaire (WPAIQ) [25]
  o Questions related to the effect your health issues have on work and regular activities
- EuroQol 5 Dimensions 5 Level Questionnaire (EQ-5D-5L) [26]
  o Two-part questionnaire with the EQ-5D descriptive system and the EQ visual analogue scale to assess patients’ health state in five dimensions: mobility, self-care, usual activities, pain/discomfort and anxiety/depression
- Capability Questionnaire (ICECAP-A) [27]
  o Capability instrument for adults

The baseline and follow-up questionnaire differ where:

1. The baseline questionnaire contains text prior to the EHP-30 as follows “This questionnaire asks about ‘symptoms due to endometriosis.’ We realise that you do not know whether or not you have endometriosis so please try and ignore the references to endometriosis and simply answer the questions focusing on the symptoms.”
2. The questions in the MYMOP2 questionnaire are adapted in the follow-up questionnaires to account for the fact that the participant needs to remember how they answered this questionnaire at baseline.

### Trial follow-up outcome form

The trial follow-up outcome form documenting analgesic use, hormonal medication, pregnancy events and use of healthcare services will be collected at 3, 6 and 12 months post-operatively via telephone by the research team. Participants will also be asked about any impacts associated with their condition on their work, caring responsibilities or other usual activities (e.g. education).

### Surgical case report form

Following laparoscopy, the surgeon will complete a surgical case report form (SCRF) detailing information about the operation. For all participants this will be:

- Grade of surgeon and whether they self-identify as an advanced laparoscopic surgeon
- Diagnosis of endometriosis subtype (SPE, ovarian endometrioma, deep)
- Ovarian cyst present that requires removal
- Confirmation of eligibility to be randomised
- Details of SPE (location, number and appearance of lesions)
- Details of other findings (fibroids, adhesions, peritoneal pockets)
- Hysteroscopy/cystoscopy taking place during laparoscopy (yes/no)
- Pathology results if endometriosis is removed or deep disease or endometrioma found

For all randomised participants:

- Details of any tubal dye tests performed
- Duration of surgery
- Concurrent intrauterine system/device insertion
- Number and location of accessory ports, not including optical port.
- Intraoperative complications (e.g. uterine perforation, anaesthetic complication, injury to surrounding anatomy, haemorrhage, and laparotomy)
- Details of images captured for diagnosis

For randomised participants allocated to surgical removal of SPE:

- Type of removal will be recorded (excision, ablation or combined)
- Details of images captured showing any removal
- Subjective assessment of whether or not complete removal was achieved (and reasons for this)
- Histological result if SPE excised and sent for histological assessment

The SCRF includes a specific set of pre-determined images. For the diagnostic laparoscopy, this is a standard panel of images of the pelvis which include the uterovesical fold, pouch of Douglas and right and left ovarian fossa. If allocated to surgical removal, the type of surgical removal will be documented, and an assessment of the adequacy of removal and images of treated areas will be performed. Three independent clinicians will assess these images, blind to operating surgeon’s classification, to reduce the likelihood of misclassification and complete an electronic surgical verification form detailing extent of endometriosis and adequacy of removal (if carried out).

### Post-operative diary

Participants will be asked to complete a diary of analgesic use and numerical rating score for worst and average pelvic pain on days 1 to 7 post-operatively.

### Post-operative form

A post-operative form (part of the electronic data collection form (DCF) will be completed detailing complications up to 30 days post-operatively. This will be completed by the clinical research team, utilising information gained by a phone-call to the patient and correlated with the participant’s hospital record. Specific complications include urinary retention, unintended overnight stay, haemorrhage, transfusion, pelvic haematoma, visceral injury (bowel, bladder, ureteric), infection (urinary, chest, wound, pelvic abscess, other), venous thromboembolism, fistula, hernia, return to theatre, readmission, ITU admission and death. Any reportable adverse events (AE) will be collected up to 30 days post-operatively.

### Data collection form

At baseline, the following personal data will be collected from trial participants in the data collection form (paper or electronic via a secure web interface to the trial database): name, NHS number or community health index number (Scotland only), unique hospital number, email address, telephone number, home address, ethnicity and year of birth. On the day of randomisation, an electronic DCF will be completed by a member of trial team and will include current analgesic use, current hormone use, visits to primary and secondary care providers, postcode to determine deprivation index, clinical pregnancy test result, future fertility intent, occupation and education, presence of dysmenorrhea (yes/no), presence of dyspareunia (yes/no) and presence of cyclical pain without menses (yes/no).

### Plans to promote participant retention and complete follow-up {18b}

Participant retention strategies include flexibility in data collection method and timing. Participants can provide data in person or using telephone, online or postal methods with a 2–4-week window provided around each point of follow-up. Support is available from a clinical research nurse via telephone if a participant may experience distress when completing the trial questionnaires.

### Data management {19}

Electronic data will be stored on University of Edinburgh secure server which is password protected and access is limited according to trial role. The participant’s email address will be kept securely on the trial database for the purposes of follow-up. Data from the trial composite questionnaire, trial follow-up outcome form (DCF), post-operative form, and SCRF will be uploaded and stored on a secure database. Personal data will be stored for a minimum of 5 years by the research team in a locked cabinet with limited access in research offices in all centres. Surgical digital images will be anonymised and uploaded. If print out images are only available, these will be scanned and then uploaded via the secure web interface.

The University of Edinburgh and NHS Lothian are joint data controllers. Any data breaches will be reported to the University of Edinburgh and NHS Lothian Data Protection Officers who will onward report to the relevant authority according to the appropriate timelines if required.

### Confidentiality {27}

All evaluation forms, reports and other records will be identified in a manner designed to maintain participant confidentiality. All records will be kept in a secure storage area with limited access. Clinical information will not be released without the written consent of the participant. The investigator and trial site staff involved with this trial may not disclose or use for any purpose other than performance of the trial, any data, record or other unpublished information, which is confidential or identifiable, and has been disclosed to those individuals for the purpose of the trial. Prior written agreement from the sponsor or its designee must be obtained for the disclosure of any said confidential information to other parties.

### Plans for collection, laboratory evaluation and storage of biological specimens for genetic or molecular analysis in this trial/future use {33}

Participants consented to ESPriT2 will be offered the option of providing a venous blood sample (approximately 55 ml) for a separate biomarker study (ESPriT +). Standard processes regarding collection and processing of the samples will used by each participating site. Blood samples (serum and plasma) will be frozen and stored locally and then sent via a specialised courier to the trial management team within the University of Edinburgh. Part of the blood samples will be sent with consent to Roche Diagnostics, Penzberg, Germany, and part of the blood samples will be retained by the University of Edinburgh for future ethically approved studies. All samples will be coded and will only be identifiable via a unique participant number.

## Statistical methods

### Statistical methods for primary and secondary outcomes {20a}

Analyses will primarily be intention-to-treat where all randomised participants will be included in the analysis retained in the group to which they were allocated and for whom outcome data are available. All results will be presented as point estimates, 95% confidence intervals with associated *p*-values. We will specify all analyses to be undertaken in a statistical analysis plan to be finalised before database lock.

### Primary outcome analysis

The primary outcome, pain domain of the EHP-30 at 12 months, will be compared using a linear regression model to estimate the mean difference in outcome, including fixed effect terms for surgical removal group, baseline pain score and stratification variables. Unadjusted results will also be presented to support the findings of the primary analysis. Secondary analyses will include using repeated-measures (multi-level) models incorporating outcome data from other follow-up time points.

### Secondary outcome analysis

A similar approach to the primary analysis will be used for analyses of the other secondary outcomes, using an appropriate (depending on outcome type) regression model.

### Economic analysis

An economic evaluation will be conducted using trial data based on the primary economic outcome of cost per quality-adjusted life year (QALY) gained, with a secondary analysis of cost per clinically significant change in symptom score at 12 months (using EHP-30). The primary perspective adopted will be an NHS and personal social services perspective, but a wider societal perspective will also be pursued using trial data about impact on work productivity. The NHS resource use collected will include secondary care costs related to surgery, length of stay and complications/readmissions as well as primary care and other healthcare costs. If the trial demonstrates that laparoscopic removal is effective in the management of SPE, longer term costs and outcomes will be assessed as part of a decision-analytic model.

The primary analysis will be in the form of a cost-utility analysis using the EQ-5D-5L instrument (collected at baseline, 3 months, 6 months and 12 months) plus data on costs and resource use collected in the trial. For the cost-utility analysis, we will evaluate the cost per QALY gained at 12 months. A secondary analysis will use the EHP-30 (using the same approach as adopted for the clinical analysis) and evaluate the cost per clinically significant change in symptom scores at 12 months. For those allocated to surgical removal, the primary outcome data will be summarised by the surgical approach (excision, ablation, or combined). A range of sensitivity analyses will be conducted to explore the impact of uncertainty on the results.

### Interim analyses {21b}

For the internal pilot, we will use stop–go criteria based on a Green-Amber-Red statistical approach including sites recruiting over the first 18 months. Assuming each centre month follows an independent identically distributed Poisson distribution with mean 0.33 and an expected randomisation of 54 participants by the end of month 18, we will use the following stop–go criteria.

- ‘Green’ will be within 1 standard deviations of 54, i.e. if we have randomised 46 or more with an average rate per centre per month of 0.28 we will continue unchanged.
- ‘Amber’ will be within 1–4 standard deviations, i.e. if we randomise between 24 and 46 with an average rate per centre per month of 0.12, then we will modify (export identified best practice from best recruiting sites; and/or more sites; and/or more recruitment time).
- ‘Red’ will be if we randomise less than 24 then consideration will be given to stopping the trial, including discussions with the funder (NIHR/HTA).

## Methods for additional analyses (e.g. subgroup analyses) {20b}

### Subgroup analyses

We will perform the following sub-group analysis of the primary outcome, and test for sub-group interactions if appropriate:

- Dysmenorrhoea (yes/no)
- Dyspareunia (yes/no)
- Use of hormones (yes/no)
- Extent of disease (< 1 cm^2^, 1–3 cm^2^ and > 3 cm.^2^)
- Neuropathic pain (PainDETECT.™) defined by a score of ≥ 19

### Exploratory analyses

In addition, for those allocated to surgical removal, the primary outcome data will be summarised descriptively by the surgical approach (excision, ablation, or combined). These analyses will be exploratory only, reflecting that the modality of SPE removal is the choice of the operating surgeon rather than randomised. Consequently, the three groups (excision, ablation or combined) are likely to be imbalanced with regard to size, patient characteristics and other confounding factors. They will also be of insufficient size to draw conclusions of relative efficacy between excision and ablation.

### Methods in analysis to handle protocol non-adherence and any statistical methods to handle missing data {20c}

The trial sample size calculation accounts for a loss of 20% of primary outcome data. A sensitivity analysis using imputation of missing values will be considered only if the proportion of cases with missing values is sufficiently large.

### Plans to give access to the full protocol, participant-level data and statistical code {31c}

The datasets, including participant-level data generated during the current trial, will available via the corresponding authors 6 months following publication of the trial results.

## Oversight and monitoring

### Composition of the coordinating centre and trial steering committee {5d}

During development of the trial, a trial management group (TMG) was established to oversee and lead the trial. The TMG includes academic gynaecologists, research nurses, a clinical trials manager, a methodologist and a blinded and unblinded statistician. The TMG meets monthly. An independent TSC was established to provide oversight of trial conduct and progress. The trial steering committee meets 12 monthly and includes six members (gynaecologists, trialists and a patient and public involvement representative).

### Composition of the data monitoring committee, its role and reporting structure {21a}

An independent data monitoring and ethics committee (DMEC) of three experienced trialists and statisticians has been established to oversee the safety of participants in the trial. The DMEC will regularly review data on the outcomes and adverse events along with updates on results of other related studies and any other analyses that the DMEC may request. If the opinion of the DMEC is that one treatment is definitely more or less effective that the other, they will advise the chair of the TSC. The Trial office will forward DMEC open meeting minutes to the sponsor and funder.

### Adverse event reporting and harms {22}

Participants will be asked about the occurrence of AEs related to their surgery or related to any new medical therapies taken for treatment of their pelvic pain started from the day of laparoscopy. These will be recorded by the research teams during the 30-day follow-up phone call. No adverse events will be recorded for any pre-existing or unrelated conditions. No adverse events will be collected after 30 days post-operatively. No serious AEs will be reported to the sponsor. Any common anticipated events, e.g. surgical complications, which are collected as data on the DCF do not need to be recorded as AEs to reduce duplication of data.

### Frequency and plans for auditing trial conduct {23}

This trial is co-sponsored by The University of Edinburgh and NHS Lothian. The Academic and Clinical Central Office for Research & Development (ACCORD) sponsor representative risk assessment, if required, will determine if audit by the ACCORD quality assurance group is required. Should audit be required, details will be captured in an audit plan. Audit of investigator sites, trial management activities and trial collaborative units, facilities and third parties may be performed.

### Plans for communicating important protocol amendments to relevant parties (e.g. trial participants, ethical committees) {25}

Any changes in research activity, except those necessary to remove an apparent, immediate hazard to the participant in the case of an urgent safety measure, will be reviewed and approved by the chief investigator. Amendments will be submitted to a sponsor representative for review and authorisation before being submitted in writing to the appropriate research ethics committee and local R&D offices for approval prior to participants being enrolled into an amended protocol.

### Dissemination plans {31a}

Results from this trial will be shared through publication in a high-impact open access peer-reviewed journal publication and presented at international conferences. Results will be shared through professional bodies, such as the British Society for Gynaecological Endoscopy. Findings will be disseminated to the public via organisations such as Endometriosis.org and Endometriosis UK and published on the Chief Investigators institutional webpages (www.exppectedinburgh.co.uk).

## Discussion

This trial aims to generate high-quality data to inform clinical practice on the clinical and cost-effectiveness of surgically removing isolated SPE in women with chronic pelvic pain. Regardless of trial outcome, we believe the data collected will contribute significantly to supporting women in informed decision-making regarding their individual endometriosis treatment options.

We appreciate that there may be recruitment difficulties encountered in this trial, due to preconceived ideas about surgical outcomes amongst both patients and clinicians following surgical management of SPE, e.g. that ‘excisional’ laparoscopic surgery is curative. Similar preconceived ideas regarding treatment outcomes have been observed to act as barriers to recruitment in other surgical RCTs with placebo arms, even when evidence supporting existing surgical practice is poor [28]. However, we were reassured, during the trial planning, that most UK endometriosis surgeons recognized the poor evidence base for ablation or excision of SPE for the treatment of pelvic pain [29]. Of the surgeons who responded to our survey (*n* = 88), 81% considered a trial to be required, and 73% were willing to participate. When women with chronic pelvic pain (*n* = 1218; 98.8% of whom had endometriosis) were surveyed: 20% said they ‘definitely would’ and 26% said that they ‘would likely participate’ in our clinical trial.

We also acknowledge that we will need to recruit and consent between 2- and threefold more women (estimated to be 1000–1200) to facilitate our randomisation target of 400 women. In addition, we appreciate that we cannot control the time between consent and date of surgery (day of randomisation). Most notably, restructuring of service provision and de-prioritisation of non-emergency gynaecological operating in the UK following the COVID-19 pandemic has seen waiting times for diagnostic laparoscopy grow significantly [30, 31].

In summary, this multi-centre participant-blind parallel-group randomised controlled trial represents an important step towards the improved understanding of the role of surgical removal of SPE in the management of endometriosis-associated pain.

## Trial status

Recruitment began on 1 April 2021 and is estimated to complete on 30 January 2024. At the time of initial manuscript submission, 45 sites are recruiting, 382 participants have been consented and 89 participants have been randomised. Current protocol version is 4.0.

## Data Availability

The datasets, including anonymised patient-level data generated during the current trial, will be available via the corresponding authors 6 months following publication of the trial results. Requests will be assessed for scientific rigor prior to secure data transfer and a data sharing agreement may be required. Further requests for access to the blood sample biobank will be considered by the sponsor.

## Abbreviations

ACCORD: Academic and Clinical Central Office for Research and Development
CI: Confidence interval
BFI: Brief Pain Inventory
DCF: Data collection form
DMEC: Data monitoring and ethics committee
ECTU: Edinburgh Clinical Trials Unit
EHP-30: Endometriosis Health Profile-30
ESHRE: European Society of Human Reproduction and Embryology
ESPriT2: Effectiveness of laparoscopic removal of isolated superficial peritoneal endometriosis for the management of chronic pelvic pain in women
EQ-5D-5L: EuroQol 5 Dimensions 5 Level Questionnaire
HTA: Health Technology Assessment
ITT: Intention-to-treat
MYMOP2: Measure Yourself Medical Outcome Profile 2
NICE: National Institute of Health and Care Excellence
NIHR: National Institute of Health and Care Research
NHS: National Health Service
PCS: Pain Catastrophizing Scale
PI: Principal investigator
PUF: Pelvic Pain and Urinary Frequency (Patient Symptom Scale)
SPE: Superficial peritoneal endometriosis
TMG: Trial management group
TSC: Trial steering committee
QALY: Quality-adjusted life year
WPAIQ: Work Productivity and Activity Impairment Questionnaire
